# Morphological and Comparative Transcriptome Analysis of Three Species of Five-Needle Pines: Insights Into Phenotypic Evolution and Phylogeny

**DOI:** 10.3389/fpls.2022.795631

**Published:** 2022-02-10

**Authors:** Xiang Li, Kewei Cai, Qiushuang Zhao, Hanxi Li, Xuelai Wang, Mulualem Tigabu, Ronald Sederoff, Wenjun Ma, Xiyang Zhao

**Affiliations:** ^1^State Key Laboratory of Tree Genetics and Breeding, School of Forestry, Northeast Forestry University, Harbin, China; ^2^Southern Swedish Forest Research Centre, Faculty of Forest Science, Swedish University of Agricultural Sciences, Lomma, Sweden; ^3^Forest Biotechnology Group, Department of Forestry and Environmental Resources, North Carolina State University, Raleigh, NC, United States; ^4^State Key Laboratory of Tree Genetics and Breeding, Key Laboratory of Tree Breeding and Cultivation of State Forestry Administration, Research Institute of Forestry, Chinese Academy of Forestry, Beijing, China; ^5^College of Forestry and Grassland, Jilin Agricultural University, Changchun, China

**Keywords:** five-needle pines, *Pinus koraiensis*, *Pinus sibirica*, *Pinus pumila*, transcriptome sequencing, SSRs, *Pinus* phylogeny

## Abstract

*Pinus koraiensis*, *Pinus sibirica*, and *Pinus pumila* are the major five-needle pines in northeast China, with substantial economic and ecological values. The phenotypic variation, environmental adaptability and evolutionary relationships of these three five-needle pines remain largely undecided. It is therefore important to study their genetic differentiation and evolutionary history. To obtain more genetic information, the needle transcriptomes of the three five-needle pines were sequenced and assembled. To explore the relationship of sequence information and adaptation to a high mountain environment, data on needle morphological traits [needle length (NL), needle width (NW), needle thickness (NT), and fascicle width (FW)] and 19 climatic variables describing the patterns and intensity of temperature and precipitation at six natural populations were recorded. Geographic coordinates of altitude, latitude, and longitude were also obtained. The needle morphological data was combined with transcriptome information, location, and climate data, for a comparative analysis of the three five-needle pines. We found significant differences for needle traits among the populations of the three five-needle pine species. Transcriptome analysis showed that the phenotypic variation and environmental adaptation of the needles of *P. koraiensis*, *P. sibirica*, and *P. pumila* were related to photosynthesis, respiration, and metabolites. Analysis of orthologs from 11 *Pinus* species indicated a closer genetic relationship between *P. koraiensis* and *P. sibirica* compared to *P. pumila*. Our study lays a foundation for genetic improvement of these five-needle pines and provides insights into the adaptation and evolution of *Pinus* species.

## Introduction

The five-needle pines are important commercial tree species, belonging to the genus *Pinus* in the Pinaceae family. They are key components of coniferous and broad-leaved mixed forest (Liu and Biondi, 2020; Goeking and Windmuller-Campione, 2021). There are 28 five-needle pine species, mainly distributed in Eurasia and North America, of which 12 five-needle pines are found in China. Only three pine species inhabit northeast China, which are *Pinus koraiensis*, *Pinus sibirica*, and *Pinus pumila* (Yang, 2014). *Pinus koraiensis* is distributed in Changbai, Zhangguangcailing, Laoyeling, Wanda, and Xiaoxinganling Mountain areas. Its northernmost boundary is Shengshan Forest Farm in Aihui County, Heihe City (Wu et al., 2021a,b). *Pinus sibirica* (Siberian pine) is only distributed in Altai, Xinjiang, and Daxinganling, Inner Mongolia. *Pinus pumila* (Siberian dwarf pine) only found in high-altitude mountain regions such as Daxinganling, Xiaoxinganling, Zhangguangcailing, and Changbai Mountains, scattered in small communities (Yang et al., 2021). These species show zonal distribution with variation in heat, cold, altitude, latitude, and longitude. They have commonalities and specificity in morphology and distribution. For instance, the external morphology and wood properties of *P. koraiensis* and *P. sibirica* are very similar, and it is difficult to distinguish them before seed production. Compared with *P. koraiensis*, *P. sibirica* had stronger cold resistance (Wang et al., 2019), while the natural distributions overlap. *Pinus pumila* is a perennial evergreen shrub with no vertical types observed. Its natural distribution overlaps with *P. koraiensis* and *P. sibirica*, but the altitude extends higher than that of *P. koraiensis* and *P. sibirica*. Furthermore, there is no reproductive isolation between the three five-needle pines species, and hybrid progeny have been obtained through interspecific hybridization (Goroshkevich et al., 2013). Although there are obvious differences between the cones and pine nuts of the three five-needle pine species, some scholars believe that *P. koraiensis*, *P. sibirica*, and *P. pumila* are just closely-related species (Dong et al., 2016).

Compared with other Pinaceae species, five-needle pines only contain one vascular bundle in the cross section of needles, and they are considered to be a relatively primitive and ancient species, which is important for studies of the origin and evolution of the Pinaceae (Liu et al., 2005). In addition, *Pinus* species are characterized by long life spans, and high levels of genetic diversity (Alicandri et al., 2020; Zeb et al., 2020). There are few reports on the genetic relationships of *Pinus* species, especially the five-needle pines (Niu et al., 2013; Baker et al., 2018; Zhao et al., 2018). The genetic relationships of *P. pumila* (Pall) Regel f. hingganensis, *P. pumila* and *P. sibirica* in the Daxinganling Mountains were examined based on peroxidase isoenzyme variation, and significant differences among the three five-needle pines was observed, and the genetic relationship between *P. pumila* and *P. sibirica* is relatively close (Chen et al., 1997). Using the same molecular makers, another study concluded that *P. koraiensis*, *P. pumila*, and *P. sibirica* were three distinctly different species (Shao et al., 2000). Following the development and application of DNA molecular markers and genome wide surveys, some studies explored the genetic relationship between *P. koraiensis P. pumila*, and *P. sibirica* using simple sequence repeats (SSR), and other markers of the nuclear, chloroplast, and mitochondrial genomes (Liu et al., 2005; Syring et al., 2005; Ayijiamali et al., 2013). There was obvious genetic differentiation among the three five-needle pines, but the genetic relationships among the three five-needle pines in Northern China was not clarified. Due to different genetic markers and research purposes, previous studies focused on the identification of *Pinus* species according to specific type of molecular marker, which cannot systematically identify the evolutionary relationships of species. Comparative transcriptome and ortholog analysis can play a crucial role in studies of plant evolution and can be used to explore genetic relationships of related species (Yang et al., 2017; Hu et al., 2018). At the moment, doubt remains about the genetic relationships among the three five-needle pines species as there is no genomic evidence to support this proposal. Clarifying the genetic relationships and taxonomy among these three five-needle pines would help us to understand the floral composition, origin and evolution of the northeast forest region of China, and would aid in the protection of these germplasm resources.

In this study, we compared the needles variation and transcriptome pattern of three endemic five-needle pines from different regions of northern China. In general, leaf morphological such as shape and size are considered as an important index for analyzing phenotypic genetic variation, which can reflect the adaptability of plants to different habitats and conditions (Guo et al., 2018). Expressed sequence tag-simple sequence repeat markers (EST-SSRs) were also identified and compared in these three five-needle pines. We also investigated the gene-environment correlation of the three species. A total of 11 pine species, including two, three, and five-needle pines were compared to infer evolutionary relationships using transcriptome sequences. These results will contribute to greater understanding of the genetic differentiation and relationships of the three five-needle pines in the *Pinus* phylogeny.

## Materials and Methods

### Plant Materials

A total of 172 tree samples from six natural populations of three five-needle pines [*P. pumila* (Pall.) Regel, *P. sibirica* (Du Tour), and *P. koraiensis* (Siebold and Zuccarini)] were collected at the mature stage of needles development and used to document phenotypic variation (Table 1). The collected samples were divided into three groups according to the species, and each species has two representative populations including *P. pumila* (ALS and LJ), *P. sibirica* (MG and IKT), and *P. koraiensis* (LS and MES). The altitude of the samples ranged from 231 to 2,052 m. Upon collection, the needles were quickly chilled and held at 4°C in a refrigerator for phenotypic measurement. For each individual, three needles were considered as one mixed sample. Four needle traits were measured, needle length (NL), needle width (NW), needle thickness (NT), and fascicle width (FW). The measurement methods were described in a previous study (Liu et al., 2021). For each species, fresh needle samples were quickly frozen in liquid nitrogen and stored at −80°C refrigerator for RNA extraction and transcriptome sequencing.

**Table 1:** Summary of sampled populations of three *Pinus* species.

Species	Population	Location	Sample size	Latitude (N)	Longitude (E)	Altitude (m)
*Pinus pumila*	ALS	Genghe, China	30	51°48'56.38''	122°00'52.66''	1,326.5
	LJ	Linjiang, China	30	41°42'05.39''	127°50'09.89''	2,052
*Pinus sibirica*	MG	Genghe, China	30	52°14'07.03''	121°46'27.85''	703.4
	IKT	Irkutsk, Russia	22	52°50'02.30''	105°54'16.63''	667
*Pinus koraiensis*	LS	Yichun, China	30	47°43'48''	128°55'12''	231
	MES	Shangzhi, China	30	45°16'22''	127°30'14.40''	536

### Collection of Climate Data

To better understand the relationship of species distribution and climate factors, the climate data was downloaded from the WorldClim database.^1^ The data package was at a resolution of 2.5 arc minutes (about 5 km^2^), and the climate data was observed from 1950 to 2000. The climate data was imported into the DIVA-GIS software (v.7.5).^2^ According to the geographic coordinates of the natural populations of the three five-needle pines, 19 bioclimatic variables (bio1 to bio19) including temperature and precipitation, for each population, were obtained. The bioclimatic variables, from bio1 to bio19, were annual mean temperature, diurnal temperature difference, isothermality, seasonal variance of temperature, extreme high temperature, extreme low temperature, temperature year difference, mean temperature in the wettest season, mean temperature in the most dry season, mean temperature in the warmest season, mean temperature in the coldest season, annual precipitation, precipitation in the wettest month, precipitation in the most dry month, variance coefficient of seasonal precipitation, precipitation in the wettest season, precipitation in the most dry season, precipitation in the warmest season, precipitation in the coldest season, respectively. The heatmap and cluster analysis of needle traits and climate variables were performed using TBtools (Chen et al., 2020). The correlation analysis of the needle traits and climate factors of different populations of the three five-needle pines was performed with the Corrplot (v. 0.84) package in R.

### Identification of SSRs

The assembled transcriptome data from *P. koraiensis*, *P. sibirica*, and *P. pumila* in this study was employed to identify EST-SSR markers. A total of 58,526, 64,961, and 57,855 unigenes were used to detect the EST-SSRs of *P. koraiensis*, *P. pumila*, and *P. sibirica* using the Simple Sequence Repeat Molecular Marker Developer (SSRMMD),^3^ respectively (Table 2). The parameters for identifying SSR motifs were set with the minimum repeats of 10 for mononucleotides, seven for di-nucleotides, six for tri-, five for tetra-, four for penta-, and four for hexa-nucleotide motifs. Because of the artifactual biosynthesis of mononucleotide repeats during PCR amplification, the mononucleotide class was removed from the SSR distribution plot.

**Table 2:** Transcriptome sequences from 11 *Pinus* species.

Species	Number of needles per fascicle	Data source	Number of unigenes	Total assembled bases (bp)	Mean length (bp)	GC content (%)	Contig N50 (bp)
*Pinus koraiensis*	Five	Illumina sequencing	58,526	43,077,328	736	43.66	1,190
*Pinus pumila*	Five	Illumina sequencing	64,961	59,629,185	917	43.11	1,561
*Pinus sibirica*	Five	Illumina sequencing	57,855	55,416,491	957	42.90	1,662
*Pinus armandii*	Five	NCBI	59,096	49,853,441	843	41.24	1,614
*Pinus squamata*	Five	NCBI	32,707	34,243,002	1,046	41.85	1,841
*Pinus parviflora*	Five	NCBI	32,314	32,282,129	999	42.30	1,796
*Pinus morrisonicola*	Five	NCBI	70,078	51,717,651	738	43.60	1,481
*Pinus yunnanensis*	Three	NCBI	74,407	57,899,050	778	42.44	1,394
*Pinus elliottii*	Two or three	NCBI	43,454	42,227,373	971	42.14	1,898
*Pinus tabuliformis*	Two	NCBI	58,530	49,833,986	851	42.35	1,550
*Pinus massoniana*	Two	NCBI	134,048	82,033,458	611	41.61	984

### Transcriptome Sequencing and Data Processing

Total RNA was extracted from the needle samples using the RNAprep Pure Plant Kit (DP441, Tiangen). The integrated cDNA libraries were constructed using the methods described by Li et al. (2021). The high-quality cDNA libraries were paired-end sequenced using a 2 × 150 bp run on an Illumina HiSeq 2500. For quality control, the raw sequencing reads were processed and checked by fastp (version 0.12; Chen et al., 2018).^4^ Because of the lack of a reference genome, the clean data were used for *de novo* transcript assemblies using Trinity (v2.11.0) for each species (Grabherr et al., 2011). To remove redundant transcripts CD-HIT (v4.8.1) was applied (Li and Godzik, 2006; Fu et al., 2012). The TransDecoder tool^5^ was then employed to predict coding sequences (CDS) from the assembled transcripts. The longest transcript of the isoforms was considered as a unigene for comparative transcriptomic and phylogenetic analysis. The functional annotation of unigenes from each species was based on the uniport^6^ and pfam^7^ databases. Based on the common protein ID, differential expression analysis of the three five-needle pine species was performed using the DEseq2 R package (v1.26.0; Love et al., 2014). Gene Ontology (GO) enrichment of differentially expressed genes (DEGs) was carried out using Omicsmart tools.^8^

### Orthologs and Phylogeny of Eleven *Pinus* Species

Orthologs are set of homologous genes that have the same common ancestral gene after speciation (Koonin, 2005). The transcriptome data of the other eight pine species was obtained from the National Center for Biotechnology Information (NCBI). The accession numbers are: *Pinus armandii* (SRR13823587), *Pinus squamata* (SRR13823429), *Pinus parviflora* (SRR13823491), *Pinus morrisonicola* (SRR13823506), *Pinus yunnanensis* (SRR13823627), *Pinus elliottii* (SRR13823584), *Pinus tabuliformis* (SRR13823418), and *Pinus massoniana* (SRR13823530). All of the Illumina sequence data for each species was independently assembled using Trinity. The assembled sequences were screened to remove redundancies and then clustered using CD-HIT (v4.8.1). The CDS were predicted by Transdecoder. To identify the evolutionary relationships of the *Pinus* species, the orthologs of proteins from 11 species were compared using OrthoFinder (Emms and Kelly, 2015) based on sequence similarity. The parameters were: “-inflation 1.5.,” the phylogenetic tree construction was done with FastTree, and multiple sequences were aligned using DIAMOND (Buchfink et al., 2015).

## Results

### Phenotypic Differentiation of Needle Traits of Three Five-Needle Pines

**Figure 1:**
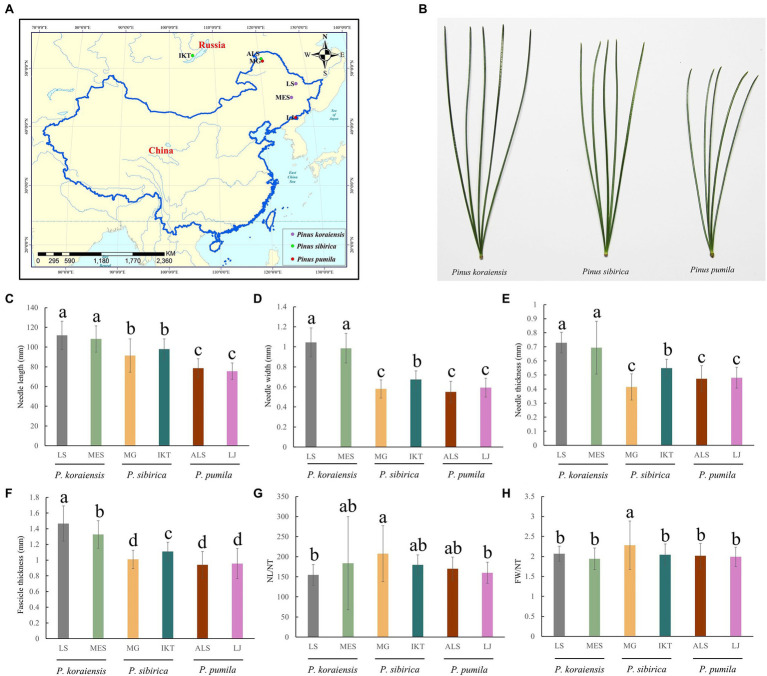
Variation of needle traits in natural populations of three five-needle pines. **(A)** Location of three five-needle pines samples collected in this study. **(B)** Phenotypes of the three five-needle pines in natural conditions. **(C)** Needle length (NL). **(D)** Needle width (NW). **(E)** Needle thickness (NT). **(F)** Fascicle width (FW). **(G)** NL/NT. **(H)** FW/NT. All data were given as means ± SE. The different lowercase letters above the histogram bars represents significant differences across the two panels (*p* < 0.05). One-way ANOVA was used to generate the *p* values. The x-axis represents the populations of *Pinus pumila* (ALS and LJ), *Pinus sibirica* (MG and IKT), and *Pinus koraiensis* (LS and MES). The y-axis indicates the phenotypes. NL, needle length; NW, needle width; NT, needle thickness; and FW, fascicle width.

The three five-needle pines possessed specific genetic backgrounds and natural distributions, which resulted in their phenotypic differentiation and climatic adaptation. Analysis of phenotypic variation was carried out to quantify the variation of needle traits. Around 172 needle samples from six natural populations (MG, LJ, ALS, IKT, LS and MES; Table 1; Figure 1A) were collected in present study, which showed clear differences (Figure 1B). The needles length of *P. koraiensis* was the longest with a mean of 110 mm, followed by *P. sibirica* (94.7 mm), and *P. pumila*, which was the shortest (77.7 mm), showing significant genetic variation (*p* < 0.05). In addition to NL/NT and FW/NT, there are significant differences between other needle traits among populations of the three five-needle pine species (*p* < 0.05; Figures 1C–H; Supplementary Table S1).

### Correlation of Needle Traits and Climate Factors

**Figure 2:**
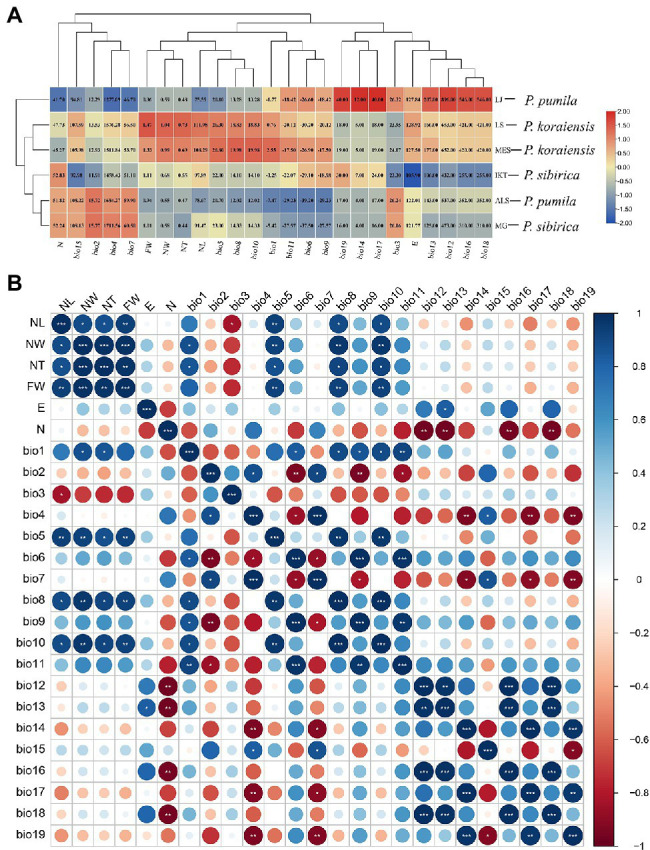
Correlation between needle traits and climate factors in the three five-needle pines. **(A)** Heatmap and cluster analysis of needle traits and climate factors in natural populations of *Pinus koraiensis*, *Pinus sibirica*, and *Pinus pumila*. The number in heatmap indicates the specific size of variables. **(B)** Correlation analysis of 25 characteristic related to needle traits and climate factors. The analysis was performed using R software. One asterisk (*) indicates the *p* < 0.05; two asterisks (**) means *p* < 0.01; and three asterisks (***) means *p* < 0.001. The circle from small to large indicates the corresponding correlation coefficient from low to high. The color scale of red and blue refers to negative correlations and positive correlations, respectively. NL, needle length; NW, needle width; NT, needle thickness; FW, fascicle width; E, longitude; N, latitude; bio1, annual mean temperature; bio2, diurnal temperature difference; bio3, isothermality; bio4, seasonal variance of temperature; bio5, extreme high temperature; bio6, extreme low temperature; bio7, temperature year difference; bio8, mean temperature in the wettest season; bio9, mean temperature in the most dry season; bio10, mean temperature in the warmest season; bio11, mean temperature in the coldest season; bio12, annual precipitation; bio13, precipitation in the wettest month; bio14, precipitation in the most dry month; bio15, variance coefficient of seasonal precipitation; bio16, precipitation in the wettest season; bio17, precipitation in the most dry season; bio18, precipitation in the warmest season; and bio19, precipitation in the coldest season.

To explore the correlations between needle traits and climatic factors, heatmap, cluster, and correlation analyses were performed. The climate data for the six populations of the three five-needle pines was obtained from the WorldClim database using DIVA-GIS as shown in Figure 2A and Supplementary Table S2. The heatmap suggested that the *P. sibirica* populations (IKT and MG) are mainly distributed in the high latitudes, where there is relatively low annual precipitation (bio12), precipitation in the wettest month (bio13), precipitation in the wettest season (bio16), and precipitation in the warmest season (bio18). For *P. koraiensis*, the climate factors, including annual mean temperature (bio1), extreme high temperature (bio5), mean temperature in the wettest season (bio8), and mean temperature in the warmest season (bio10) in its natural distribution, were higher than that for *P. sibirica* and *P. pumila*. Its average NL, NW, NT, and FW were also the largest compared with the other two five-needle pines. More importantly, these climate factors and needle traits were significantly clustered into the same subgroup in the cladogram, indicating a clear correlation. *P. pumila*, was naturally distributed in the mountains (high altitude) compared with *P. koraiensis* and *P. sibirica* (Table 1). The average values of extreme high temperature (bio5), mean temperature in the wettest season (bio8) and mean temperature in the warmest season (bio10) were all significantly lower than that for *P. koraiensis* and *P. sibirica*. Interestingly, The ALS population from *P. pumila* exhibited the lowest annual mean temperature (bio1), extreme low temperature (bio6), mean temperature in the driest season (bio9), and mean temperature in the coldest season (bio11), which indicated that *P. pumila* has strong cold resistance, and that it can survive extremely low temperatures down to minus −39°C. Four needle traits and eight temperature factors were grouped into the same subcluster.

To further understand the correlation between the needle traits of five-needle pines and climate factors, the Corrplot R package was used for correlation analysis (Figure 2B). Needle length was significantly correlated with isothermality (*p* < 0.05), extreme high temperature (*p* < 0.01), mean temperature in the wettest season (*p* < 0.05), and mean temperature in the most dry season (*p* < 0.05). There were significant correlations between the needle width and annual mean temperature (*p* < 0.05) and extreme high temperature (*p* < 0.01), mean temperature in the wettest season (*p* < 0.01), and mean temperature in the driest season (*p* < 0.01). No correlation was found between the four needle traits and precipitation as well as longitude and latitude.

### Transcriptome Sequencing and *de novo* Assembly

**Figure 3:**
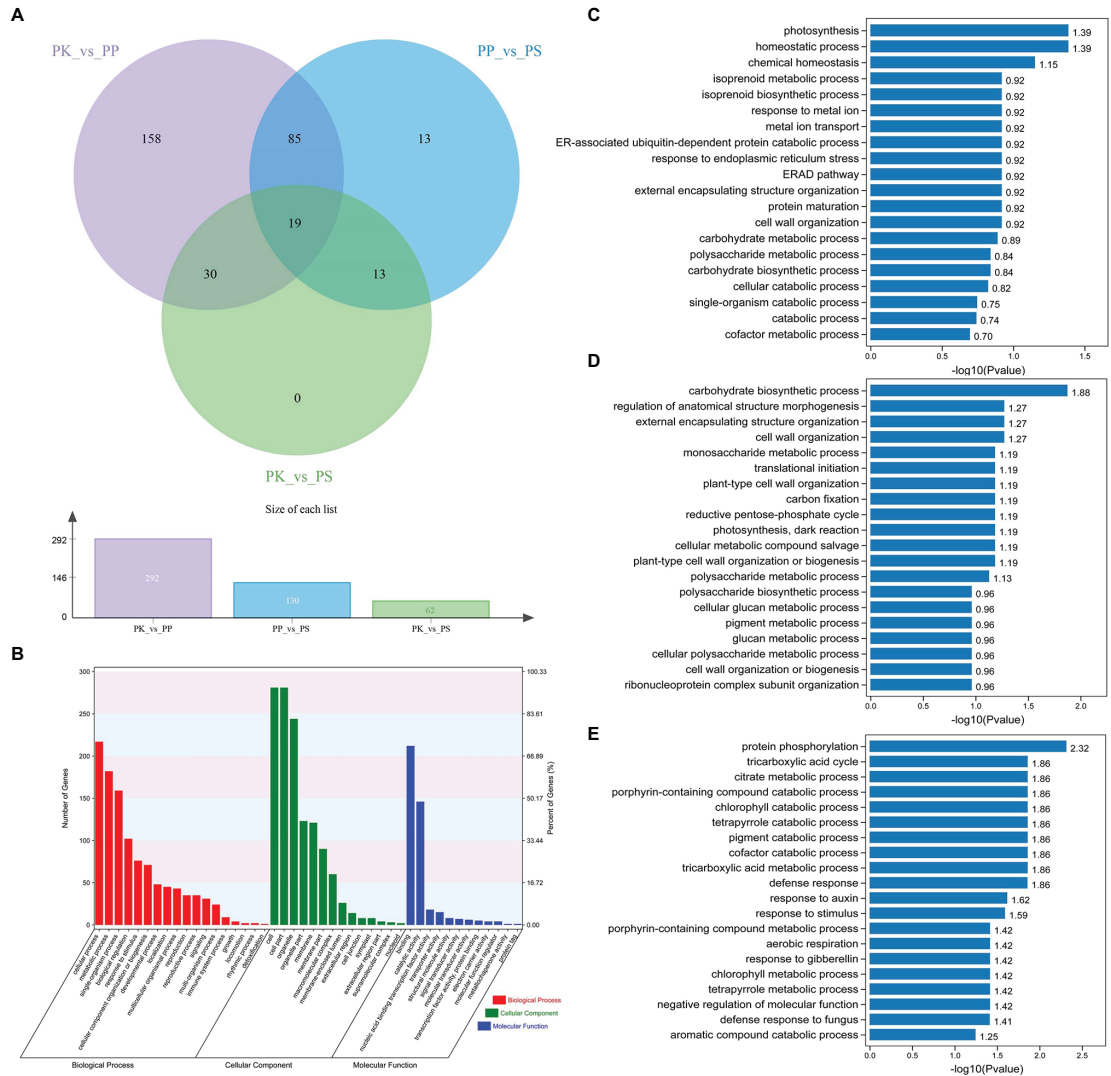
Analysis of differentially expressed genes (DEGs) in the three five-needle pines. **(A)** Venn diagram of DEGs for PK vs. PP, PK vs. PS, and PP vs. PS. **(B)** Gene Ontology (GO) analysis of DEGs for PK vs. PP, PK vs. PS, and PP vs. PS in three main categories. GO enrichment analysis of the DEGs between PK vs. PP **(C)**, PP vs. PS **(D)**, and PK vs. PS **(E)**. The x-axis indicates GO terms belonging to three categories [biological process (BP), cellular component (CC), and molecular function (MF)], the y-axis on the left represents the gene numbers for each term, while the y-axis on the right represents the percent of genes for each term.

The expression of genes in needles of the three five-needle pine species was different. Using the DEseq2 software, we investigated the DEGs of three pairs of species (PK_vs_PP, PP_vs_PS, and PK_vs_PS). From the Venn diagram, we could see that 292, 130, and 62 DEGs were identified from the PK_vs_PP, PP_vs_PS, and PK_vs_PS (Figure 3A). Nineteen genes were common to the three pairs. GO function annotation reveled that all of the DEGs were divided into 44 level-2 functional classifications, including 18 terms of biological processes (BPs), 14 terms of cellular components, and 12 terms of molecular functions (MFs). The top terms were cellular processes, metabolism, cell components (CCs), and catalytic activity (Figure 3B). To investigate more precisely the functions of the identified DEGs, GO enrichment analysis of different pairs of species was performed. For PK vs. PP, the identified DEGs were enriched in genes for photosynthesis (GO:0015979), homeostasis (GO:0042592 and GO:0048878), and isoprenoid metabolism (GO:0006720; Figure 3C). Furthermore, carbohydrate biosynthesis (GO:0016051), morphogenesis (GO:0022603), encapsulating structural organization (GO:0045229), and cell wall organization (GO:0071555) were enriched in PP vs. PS (Figure 3D). For PK vs. PS, the DEGs were enriched in protein phosphorylation (GO:0006468), the tricarboxylic acid cycle (GO:0006099), citrate metabolism (GO:0006101), and porphyrin catabolism (GO:0006787; Figure 3E).

### Identification and Distribution of SSRs

**Figure 4:**
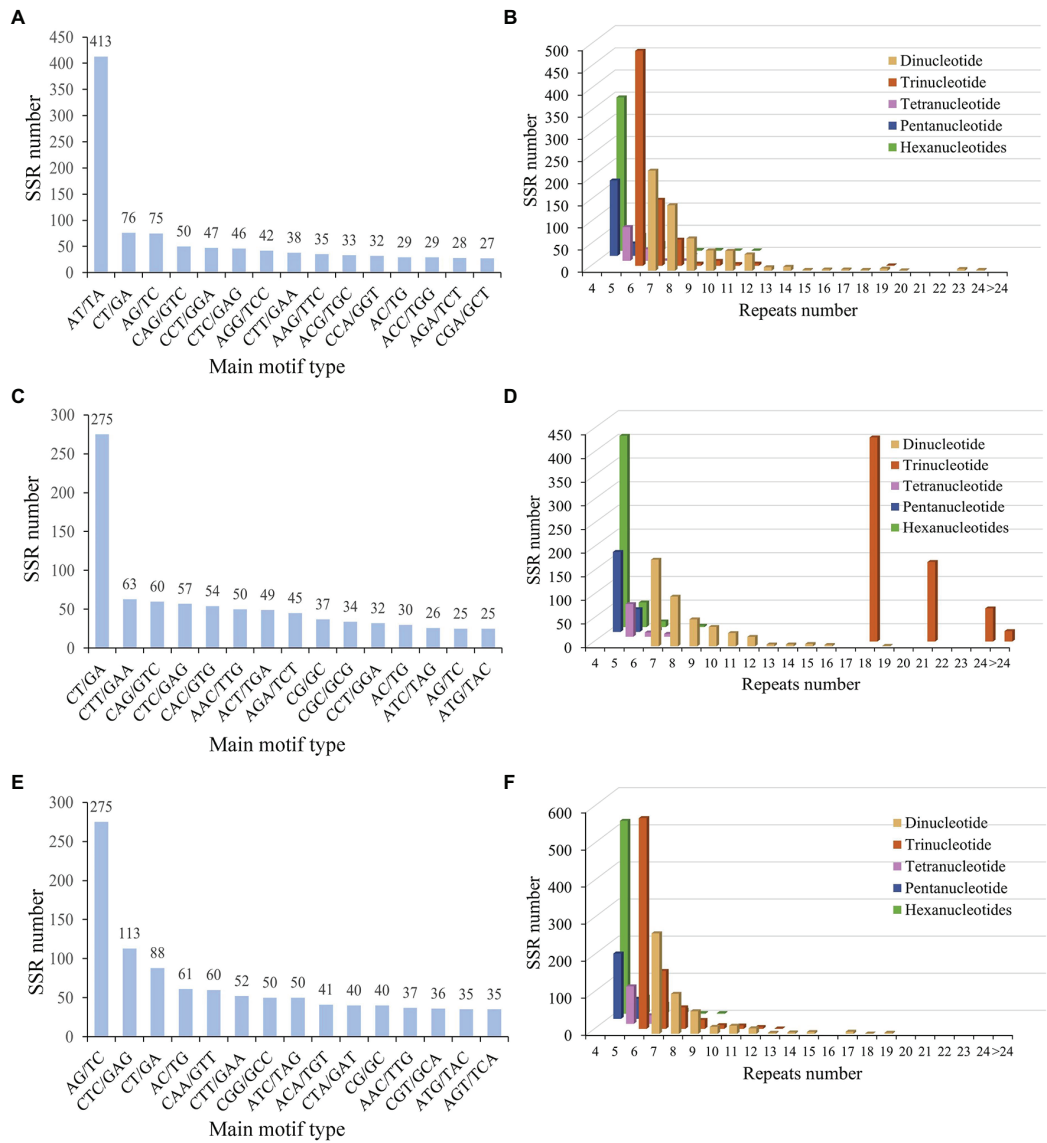
The simple sequence repeat (SSR) distribution of three five-needle pines. SSRs length distribution across different motif classes in *Pinus koraiensis* **(A)**, *Pinus sibirica* **(C)**, and *Pinus pumila* **(E)**. The number of repeats in each different motif length in *P. koraiensis* **(B)**, *P. sibirica* **(D)**, and *P. pumila* **(F)**.

Microsatellite marker is a widely distributed repeat sequence of 1–6 bases in plants with high level of genetic variation. To investigate the difference of SSRs from three five-needle pines, microsatellite marker analysis was performed, all unigenes from the *de novo* assembly were surveyed to find EST-SSRs. The number of identified unigenes with paired-end reads from *P. koraiensis*, *P. sibirica*, and *P. pumila* was 58,526, 64,961, and 57,855, with a total assembled length of 43,077,328, 59,629,185, and 55,416,491 bp, respectively. The mean length of unigenes was 736, 918 and 958 bp, with a contig N50 length of 1,190, 1,561, and 1,662 bp, respectively. Of the 4,936 (8.43%), 4,749 (7.31%), and 5,528 (9.55%) unigene sequences of *P. koraiensis*, *P. sibirica*, and *P. pumila* contained 5,410, 5,147, and 5,928 putative EST-SSRs, respectively. The most abundant motif type was AT/TA (7.63%), followed by CT/GA (1.40%), AG/TC (1.39%), CAG/GTC (0.92%), CCT/GGA (0.87%), CTC/GAG (0.85%), and AGG/TCC (0.78%; Figure 4A; Supplementary Table S3). The remaining motifs accounted for 13.16%. In addition to mononucleotide repeats, trinucleotide repeats (719, 13.29%) were the most abundant EST-SSRs in *P. koraiensis*, followed by dinucleotides (614, 11.93%) and hexanucleotides (406, 6.85%). EST-SSRs with 10 tandem repeats (31.1%) were the most frequent type, followed by 11 (12.8%), six (9.83%), four (9.54%), and seven (6.95%; Figure 4B; Supplementary Table S4). For *P. sibirica*, the dominant motif type was CT/GA (5.34%), followed by CTT/GAA (1.22%), CAG/GTC (1.17%), CTC/GAG (1.11%), CAC/GTG (1.05%), AAC/TTG (0.97%), and ACT/TGA (0.95%; Figure 4C; Supplementary Table S5). The mononucleotide, di-, tri-, tetra-, penta-, and hexa-nucleotide repeats were of 3,233 (62.81%), 451 (8.76%), 691 (13.43%), 78 (1.52%), 223 (4.33), and 471 (9.15%), respectively. Among them, the largest number of tandem repeats was 10 (33.57%), 11 (12.59%), four (11.13%), 18 (9.25%), 12 (6.02%), and seven (3.67%; Figure 4D; Supplementary Table S6). According to the microsatellite sequences of *P. pumila*, we found that the most abundant motifs of the repeat units was AG/TC (4.64%), followed by CTC/GAG (1.91%), CT/GA (1.48%), AC/TG (1.03%), CAA/GTT (1.01%), and CTT/GAA (0.88%; Figure 4E; Supplementary Table S7). The mononucleotide (3,615, 61.0%) motif was the most abundant, followed by trinucleotide (691, 11.7%), hexanucleotides (471, 7.95%), and dinucleotide (451, 7.61%). The number of repeated SSR units ranged from 4 to 24. Ten (33.6%) repeated units were predominant, followed by 11 (12.6%), four (11.1%), 18 (9.25%), and 12 (6.02%) tandem repeats (Figure 4F; Supplementary Table S8).

### Ortholog Identification and Functional Characterization of the Three Five-Needle Pine Species

**Figure 5:**
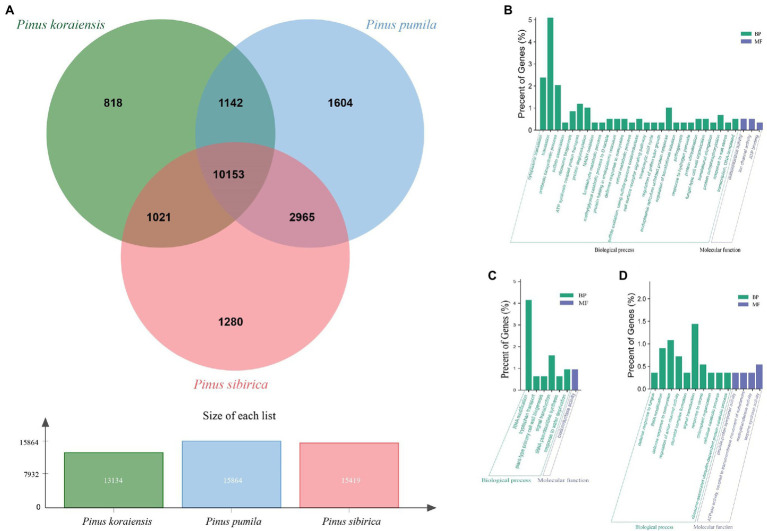
Gene Ontology enrichment analysis of orthologous gene families in the three five-needle pines. **(A)** Venn diagram depicting the shared and specific gene families in *Pinus koraiensis*, *Pinus sibirica*, and *Pinus pumila*. Orthologous clusters were evaluated for 59,412 concatenated coding sequences (CDS) shared and unique gene families. The counts in the Venn diagram represent the number of unique and shared families between *P. koraiensis*, *P. sibirica*, and *P. pumila*. Around 10,153 gene families were conserved across *P. koraiensis*, *P. pumila*, and *P. sibirica*. GO enrichment analysis of the specific gene families in *P. koraiensis* **(B)**, *P. sibirica* **(C)**, and *P. pumila* **(D)**. The x-axis indicates GO terms belonging to BPs and MFs, and the y-axis represents the percent of genes.

To further investigate the genetic variation of the three five-needle pine species, we identified unique and shared gene families and inquired about their biological functions using OrthoFinder. The Venn diagram indicates that 13,134, 15,864, and 15,419 gene families were identified among *P. koraiensis*, *P. pumila*, and *P. sibirica*, respectively (Figure 5A). Of these putative gene families, the largest number of unique gene families were found in *P. pumila* with 1,604, followed by *P. sibirica* (1,280), while *P. koraiensis* (818) possessed the lowest number of unique gene families. The common gene families between the three five-needle pines were 10,153, accounting for a large proportion of the total of 18,983 families. The number of unique gene families for each species was used and mapped to the GO database for enrichment analysis. The results were divided into three groups, (1) MF, (2) CC, and (3) BP. For *P. koraiensis*, the unique gene families were significantly enriched in translation (GO:0006412), cytoplasmic translation (GO:0002181), antibiotic biosynthetsis (GO:0017000), ATP synthesis coupled proton transport (GO:0015986), and protein deglycosylation (GO:0006517; Figure 5B). For *P. sibirica*, the families were enriched in GO terms for RNA modification (GO:0009451), signal transduction (GO:0007165), response to water deprivation (GO:0009414), oxidoreductase activity (GO:0016705), and plant-type primary cell wall biogenesis (GO:0009833; Figure 5C). In *P. pumila*, the top-eight enriched GO terms were related to signal transduction (GO:0007165), defense response to oomycetes (GO:0002229), RNA modification (GO:0009451), regulation of anion channel activity (GO:0010359), response to ozone (GO:0010193), terpene synthase activity (GO:0010333), cellulose catabolism (GO:0030245), and chloroplast organization (GO:0009658). Stomatal complex formation (GO:0010376) could also be noted, suggesting a specific function during the evolution of this species (Figure 5D).

### Phylogenetic Analyses

**Figure 6:**
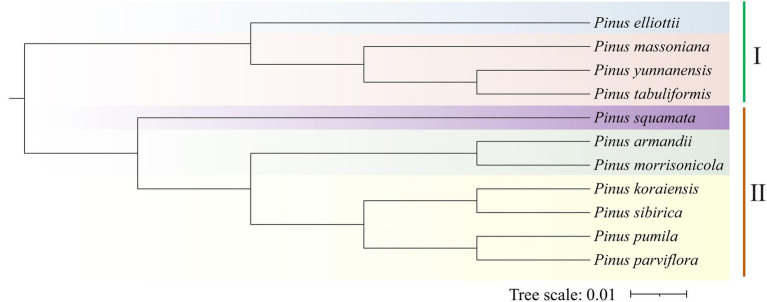
Phylogenetic relationships of *Pinus koraiensis*, *Pinus sibirica*, *Pinus pumila*, and eight other *Pinus* species. A phylogenetic tree of all 11 *Pinus* species was constructed based on orthologous genes. Two subsections of *Pinus* species are listed with Roman numerals. I represents the subsection *Pinus* for the two/three-needle pines. II indicates the subsection Strobus for the five-needle pines.

To identify the gene families and their evolution of *P. koraiensis* (18,221 proteins), *P. sibirica* (19,337 proteins), and *P. pumila* (21,854 proteins) and other eight *Pinus* species, a comparative analysis of the orthologous sets was carried out (Table 3). The public transcriptome data of the other eight confer species was obtained from the NCBI database. The number of gene families in *P. massoniana* (17,367) was the highest, while *P. parviflora* (13,284) contains the lowest number of gene families between the 11 pine species. Additionally, the largest number of single copy genes was found in *P. morrisonicola* (7,082), followed by *P. massoniana* (4,673), *P. koraiensis* (3,808), *P. pumila* (3,720), and *P. yunnanensis* (2,955). To clarify the evolutionary status of the five-needle pines, phylogenetic analysis was conducted using orthologous genes. The results were in good agreement with classical taxonomy of *Pinus* species. In a phylogenetic tree, the candidate 11 *Pinus* species were grouped into two clear subsections, which consists of a two/three-needle pine group I, including *P. yunnanensis*, *P. elliottii*, *P. tabuliformis*, and *P. massoniana* and a five-needle pine group II including *P. koraiensis*, *P. pumila*, *P. sibirica*, *P. armandii*, *P. squamata*, *P. parviflora*, and *P. morrisonicola* according to the orthologous genes (Figure 6). Of these species, the three five-needle pines (*P. koraiensis*, *P. sibirica*, and *P. pumila*) of our interest were clustered, showing a close relationship. *P. koraiensis* and *P. sibirica* possess a high level of genetic relatedness, both showing a tall tree form, while they specifically separate from *P. pumila* (showing a shrub like form) at the species level.

**Table 3:** Statistical information of proteins, clusters, and singletons in 11 genus *Pinus* species.

Species	Proteins	Clusters	Singletons
*Pinus koraiensis*	18,221	13,487	3,808
*Pinus pumila*	21,854	16,737	3,720
*Pinus sibirica*	19,337	15,523	2,632
*Pinus armandii*	19,532	15,517	2,833
*Pinus squamata*	17,259	14,210	1,866
*Pinus parviflora*	15,741	13,284	1,530
*Pinus morrisonicola*	27,302	16,383	7,082
*Pinus yunnanensis*	20,826	16,786	2,955
*Pinus elliottii*	17,626	14,664	2,221
*Pinus tabuliformis*	18,514	15,528	2,119
*Pinus massoniana*	23,290	17,367	4,673

## Discussion

### Phenotypic Variation and Environmental Adaptation of Three Five-Needle Pines

Plant leaf/needle traits have phenotypic plasticity due to climate change (Asao et al., 2020). Previous studies found phenotypic traits of *P. koraiensis* differed significantly between and within different populations (Tong et al., 2019). Also, the leaf fresh weight, dry weight, and moisture content of trees in different populations of *P. pumila* show significant (*p* < 0.01) differences (Wei et al., 2018). However, the relationship between genetic variation and environmental adaptation for needle traits among *P. koraiensis*, *P. sibirica*, and *P. pumila* was still unclear. In the present study, there are significant differences in needle length and needle width among these three five-needle pines. The needle length of *P. koraiensis* was the longest, followed by *P. sibirica* and *P. pumila*, which may be related to their specific geographic distribution during recent evolution. In the natural range of *P. koraiensis*, high precipitation and high temperature occur in the summer, forming suitable climatic conditions, for its needle growth and development. A high annual mean temperature and extreme high temperature were also found. In contrast to *P. koraiensis*, *P. sibirica*, and *P. pumila* are mainly distributed in alpine or high latitude areas, characterized by relatively low temperatures, which may be limiting factors for the species distribution. Correlation analysis also confirmed these results and indicated that the genetic variation of needle traits significantly correlated with temperature. Although *P. sibirica* and *P. pumila* can grow in the regions with relatively low temperature, *P. pumila* was mainly distributed in the alpine areas with high precipitation and high average latitude of 1,689 m. Thus, climate factors, especially temperature and altitude, may appear to be the main factors controlling the growth and development of needles and thereby determine the species distribution of *P. koraiensis*, *P. sibirica*, and *P. pumila*.

### The Mechanisms of Adapting to Habitat Conditions

Although plants grow in specific geographical environments, their physiology and metabolism need to take place under appropriate temperature, light, water, and rhizosphere environments. To further understand the regulatory mechanisms controlling the variation of needles in these three five-needle pines, transcriptome analysis was performed.

**Figure 7:**
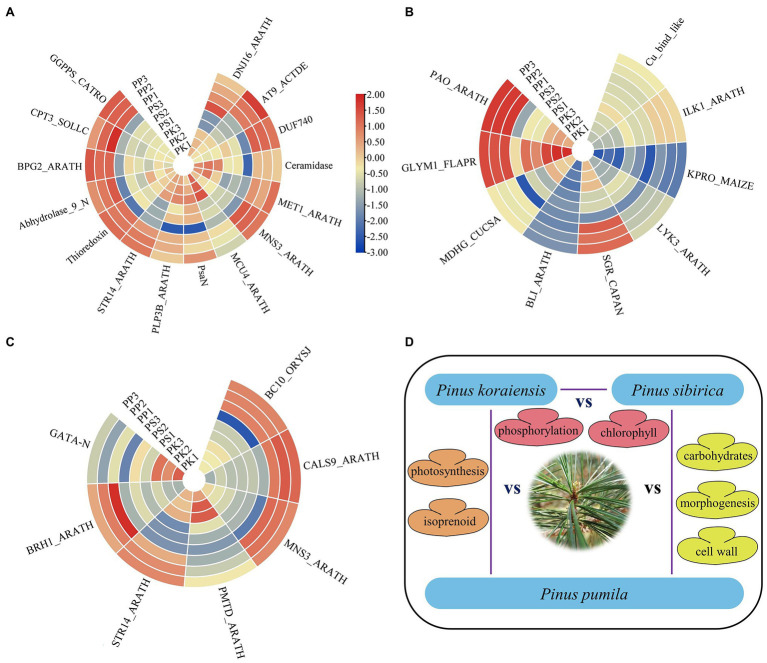
The expression heatmap of DEGs enriched in the first four GO terms. **(A)** The heatmap of enriched DEGs in PK_vs_PP. **(B)** The heatmap of enriched DEGs in PK_vs_PS. **(C)** The heatmap of enriched DEGs in PS_vs_PP. **(D)** Schematic diagram of the mechanism of needle adaptation.

In the present study, the DEGs of PK_vs_PP specifically were assigned to photosynthetic and homeostatic terms in GO enrichment analysis, suggesting that the DEGs involved in needle morphogenesis of *P. koraiensis* and *P. pumila* participate in complex metabolic processes. Previous studies have confirmed that light intensity, photoperiod and light composition affect many characteristics of plant development (Zhang et al., 2019; Jan et al., 2021). The GO analysis indicates a role for genes acting in isoprenoid metabolism, which regulates primary and secondary metabolism in plants (Lange et al., 2020; Mahmoud et al., 2021). The correlation may be the result of plant adaptation to the environment during evolution (Pulido et al., 2012), possibly to plant-environment interactions (Tadjouate et al., 2021). The upregulated DEGs in the needles of *P. pumila*, may therefore be related to its adaptation to an alpine habitat, determining needle growth and development (Figure 7A; Supplementary Table S9).

*Pinus koraiensis* and *P. sibirica* are five-needle pines (subgroup Strobus) of the single vascular bundles with similar morphology. In this study, the GO enrichment analysis of DEGs between *P. koraiensis* and *P. sibirica* showed that these genes are mainly related with primary metabolism through the tricarboxylic acid cycle, citrate metabolism, and porphyrin catabolism and involved in respiration and chlorophyll formation. The expression of one gene (SGR_CAPAN) in *P. koraiensis* was higher than that in *P. sibirica*, and it mainly triggers chlorophyll degradation during leaf senescence (Figure 7B). The distribution pattern of chlorophyll content in needles and response to water stress of *P. koraiensis* and *P. sibirica* display significant differences, supporting the results reported here (Ayijiamali et al., 2013). In addition, The GO terms “protein phosphorylation” were also identified in enrichment analysis of PK vs. PS. Protein phosphorylation plays an important role in plant signal transduction, epigenetic regulation, and growth and development (Li et al., 2020). The heatmap and correlation analysis from needle traits and climate data indicated that there are relatively high temperatures and high precipitation in natural populations of *P. koraiensis* compared with that of *P. sibirica*, perhaps related to leaf development.

*Pinus sibirica* and *P. pumila* are important conifer trees in Daxinganling Mountains of China. Presumably, owing to the difference in altitude, the morphology and chemical composition of the needles show clear differentiation. Although the needle length of *P. pumila* was the lowest in the present study, *P. pumila* possesses strong low temperature tolerance compared with *P. sibirica*, which may be related to biosynthesis of cryoprotective compounds, similar to results previously reported (Jin et al., 1998). Carbohydrate biosynthesis was a major GO term in PP vs. PS, presumably due to the involvement of starch and sugar in leaf/needle development. Similar results were also found in other plants such as *Vitis vinifera* (Gao et al., 2021), *Hevea brasiliensis* (Gersony et al., 2020), and *Pinus thunbergia* (Zhang et al., 2020). Additionally, regulation of morphogenesis, organization of external encapsulating structures and cell wall biosynthesis GO terms were identified, which may be related to the difference in stem morphology (apical dominance) between *P. sibirica* and *P. pumila*. *Pinus sibirica* is a perennial tall tree, while *P. pumila* is a creeping shrub, and to date, there is no erect type for this species. Therefore, we presume that the DEGs are probably involved in plant morphogenesis including stem and leaf development, especially the formation of cell wall organization (such as *BC10_ORYSJ* and *MNS3_ARATH*) as they show high expression on the heatmap (Figure 7C). Similar results were also found in the GO enrichment of gene families of *P. pumila*, especially for cellulose catabolism. One gene (*BRH1_ARATH*), involved in the brassinosteroid (BRs) signaling pathway was identified, which specifically regulates the growth and development of rosette leaves and can be a candidate target gene for needle development (Figure 7D).

### Identification of Evolutionary Status

Owing to ecological and economical values, *P. koraiensis*, *P. sibirica*, and *P. pumila* were widely employed for reforestation and industrial uses. Investigating the genetic relationship and taxonomic status among these three species is significant for elucidating their evolutionary relationships and accelerating genetic improvement. Due to the limited amount of genomic information, the identification and extent of functional gene relationships of the present five-needle pines remains confusing. Transcriptome sequence has become a powerful tool for functional gene mining, estimation of genetic diversity, and identification of gene specific relationships (Estelle et al., 2021; Yu et al., 2021). Using RNA-seq technology, analysis of orthology for phylogenetic relationships and evolution between different species, have been carried out (Peng et al., 2019). In the present study, pairwise orthologs from different transcriptomes from 11 *Pinus* species were identified and used for estimation of evolutionary distance. Phylogenetic analysis showed that *P. koraiensis*, *P. sibirica*, and *P. pumila* in the present study belong to the same *Pinus* subsection (Strobus), displaying a close genetic relationship, consistent with the existing taxonomy. We observed that *P. koraiensis* and *P. sibirica* maintain relatively close genetic relationship compared with *P. pumila*. Similar results were reported in previous study of spatiotemporal evolution of global pines (Jin et al., 2021). Furthermore, in subgroup II, *P. pumila* and *P. parviflora* were divided into the same group of five-needle pines, which may correspond to the traits of short needles among them. The genetic relationships based on the EST-SSRs are highly consistent with the results of a neighbor-joining (NJ) tree inferred from chloroplast and mitochondrial DNA fragments in previous studies (Yang, 2014). As a whole, there is a close genetic relationship between *P. koraiensis* and *P. sibirica*, but their biological characteristics changed to adapt to variation in temperature, precipitation, and location.

Currently, there is limited genetic information available for the study of phenotypic variation, environmental adaptation, and evolution of these three pine species. In this study, we have systematically investigated the phenotypic and genetic differentiation of these three pine species combining morphological, climatic, and transcriptome data. These results provide new insights into gene evolution and contribute to the future genetic improvement of these three pine species.

## Data Availability Statement

The datasets presented in this study can be found in online repositories. The names of the repository/repositories and accession number(s) can be found at: https://ngdc.cncb.ac.cn/search/?dbId=&q=PRJCA006786, PRJCA006786.

## Author Contributions

XZ, RS, and WM conceived and designed the research. XL and KC performed the experiments. QZ, HL, and XW analyzed the data. XL wrote the manuscript. MT and RS revised the manuscript. XZ provided the funding. All authors contributed to the article and approved the submitted version.

## Funding

This work was funded by the Fundamental Research Funds for the Central Universities (No. 2572020AW07), the Fundamental Research Funds of Chinese Academy of Forestry (CAFYBB2020ZB003), the Innovation Project of State Key Laboratory of Tree Genetics and Breeding (Northeast Forestry University; No. 2021A01), and the Cooperative Forestry Science and Technology Project for the Zhejiang Province and Academy (No. 2021SY02).

## Conflict of Interest

The authors declare that the research was conducted in the absence of any commercial or financial relationships that could be construed as a potential conflict of interest.

## Publisher’s Note

All claims expressed in this article are solely those of the authors and do not necessarily represent those of their affiliated organizations, or those of the publisher, the editors and the reviewers. Any product that may be evaluated in this article, or claim that may be made by its manufacturer, is not guaranteed or endorsed by the publisher.

## Footnotes

^1^ http://www.worldclim.org

^2^ http://www.diva-gis.org/

^3^ https://github.com/GouXiangJian/SSRMMD

^4^ https://github.com/OpenGene/fastp

^5^ https://github.com/TransDecoder/TransDecoder/releases

^6^ https://www.uniprot.org/

^7^ http://pfam.xfam.org/

^8^ https://www.omicsmart.com/
